# Alcohol consumption and high‐molecular‐weight adiponectin levels are interactively associated with all‐cause mortality among community‐dwelling persons

**DOI:** 10.1111/acer.70037

**Published:** 2025-03-28

**Authors:** Ryuichi Kawamoto, Asuka Kikuchi, Daisuke Ninomiya, Teru Kumagi, Masanori Abe

**Affiliations:** ^1^ Department of Community Medicine Ehime University Graduate School of Medicine Toon‐City Ehime Japan; ^2^ Department of Internal Medicine Seiyo Municipal Nomura Hospital Seiyo‐City Ehime Japan

**Keywords:** alcohol consumption, high‐molecular‐weight adiponectin, Japanese, mortality

## Abstract

**Background:**

Decreased levels of high‐molecular‐weight (HMW) adiponectin are associated with metabolic syndrome and insulin resistance. This relationship may be further confounded by alcohol consumption, which plays a role in the development of liver dysfunction. In Japan, few studies have investigated the relationship between HMW adiponectin levels and alcohol consumption with mortality.

**Methods:**

The study included 845 male participants (mean age, 61 ± 13 years; range, 20–89 years) and 1065 female participants (mean age, 63 ± 11 years; range, 22–88 years). Of the participants, 809 (42.4%) were classified as nondrinkers, 561 (29.4%) as occasional drinkers, 346 (18.1%) as daily light drinkers, and 194 (10.2%) as daily heavy drinkers. A Cox proportional hazards model was used to calculate hazard ratios (HR) for all‐cause mortality, adjusting for various confounders, including HMW adiponectin levels.

**Results:**

Individuals who abstained from alcohol consumption (hazard ratio [HR], 1.23; 95% confidence interval [CI], 1.00–1.52) or engaged in daily heavy drinking (HR, 1.39; 95% CI, 1.04–1.86) exhibited significantly higher overall mortality than occasional drinkers. Additionally, those with the 3rd standard deviation (SD) level of HMW adiponectin (HR, 1.39; 95% CI, 1.07–1.80) and 4th SD level (HR, 1.65; 95% CI, 1.23–2.23) had a similarly increased risk of all‐cause mortality compared to those with the lowest levels. After adjusting for confounders, the HR for individuals with the 3rd + 4th SD levels of HMW adiponectin was significantly elevated in nondrinkers (HR, 1.89; 95% CI, 1.09–3.29), occasional drinkers (HR, 1.84; 95% CI, 1.05–3.21), and daily heavy drinkers (HR, 1.90; 95% CI, 1.05–3.44), but not in daily light drinkers. The interaction between alcohol consumption and HMW adiponectin levels was significantly associated with all‐cause mortality.

**Conclusion:**

These findings suggest that alcohol consumption and elevated HMW adiponectin levels are interactively associated with all‐cause mortality in community‐dwelling individuals.

## Graphical abstract

Alcohol consumption showed a U‐shaped relationship with all‐cause mortality, while adiponectin levels were dose‐dependently linked to mortality. Furthermore, an interaction between alcohol intake and adiponectin levels was noted, regardless of conventional cardiovascular risk factors. For a deeper understanding of the mechanisms driving this connection in healthy individuals in the community, prospective population‐based studies are necessary. These studies could also shed light on whether interventions, such as lifestyle changes that impact HMW adiponectin levels in adults, could lower mortality risk.

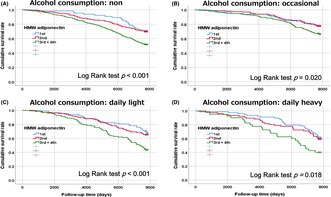

## INTRODUCTION

Plasma adiponectin, a 247‐amino‐acid protein primarily secreted by adipocytes, comprises four distinct domains that regulate glucose and lipid metabolism and insulin sensitivity (Nguyen, 2020). Adiponectin is found in the serum in three forms: a trimer (low‐molecular‐weight [LMW]), a hexamer (medium‐molecular‐weight [MMW]), and a high‐molecular‐weight (HMW) form (Pajvani et al., 2004). The HMW form has the highest receptor affinity and activates AMP‐activated protein kinase, leading researchers to conclude that HMW adiponectin possesses greater biological activity compared to LMW or MMW forms (Pajvani et al., 2004). It also enhances antiinflammatory, antiatherogenic, and antioxidant properties (Delaigle et al., 2004; Nguyen, 2020). Low levels of serum adiponectin have been identified as a risk factor for developing type 2 diabetes (Li et al., 2009), hypertension (Adamczak et al., 2003; Kim et al., 2013), metabolic syndrome (Ryo et al., 2004), and coronary heart disease (CHD) (Frystyk et al., 2007). However, recent epidemiological studies have indicated that elevated adiponectin levels are associated with increased mortality from all causes and CHD (Wu et al., 2014). Moreover, subsequent studies of similar scale have not consistently shown significant associations between adiponectin levels and CHD risk (Menzaghi & Trischitta, 2018). Thus, the current evidence remains inconclusive.

Alcohol consumption is a significant contributor to morbidity and mortality, representing approximately 5.1% of the global disease burden and 5.3% of total deaths, which leads to considerable social and economic repercussions (World Hearth Organization, 2018). Infrequent, light, and moderate alcohol consumption has been inversely associated with mortality from all causes: cardiovascular disease (CVD), chronic lower respiratory tract diseases, Alzheimer's disease, influenza, and pneumonia. Furthermore, light‐to‐moderate alcohol intake may provide protective effects against mortality linked to diabetes mellitus, nephritis, nephrotic syndrome, and nephrosis. In contrast, heavy or binge drinking correlates with an increased risk of mortality from all causes, including cancer and unintentional injuries (Tian et al., 2023). The purported benefits of low‐level alcohol consumption, especially concerning CVD mortality, have been challenged due to issues such as selection bias, reverse causation, and residual confounding, reinforcing the public health message that the safest approach to drinking is abstinence or reduced consumption (Chudzińska et al., 2022; Piano, 2017; Zhao et al., 2023).

In contrast to United States and European findings in White and Black populations that found a positive correlation between alcohol intake and serum adiponectin levels (Imhof et al., 2009; Sierksma et al., 2004), studies in Asian populations have revealed an inverse association between alcohol intake and serum adiponectin levels, suggesting ethnic and gender differences in the impact of alcohol consumption on these levels (Nishise et al., 2010). Additionally, alcohol intake does not appear to be associated with fluctuations in circulating adiponectin levels in a cohort (Bell & Britton, 2015). Therefore, the influence of alcohol consumption may further complicate the association between HMW adiponectin and all‐cause mortality, and few studies have investigated the relationship between HMW adiponectin and the development of all‐cause mortality in Japan.

This study aimed to investigate whether an interactive relationship between alcohol consumption and increased HMW adiponectin levels is independently associated with all‐cause mortality, utilizing prospective data from Japanese community‐dwelling individuals.

## METHODS

### Subjects

**FIGURE 1:**
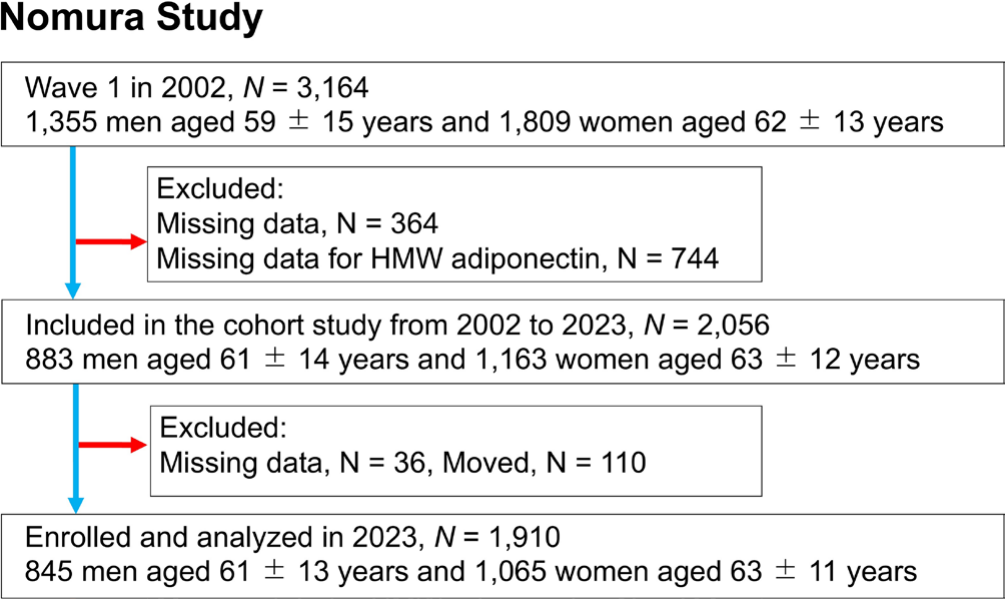
Participant flowchart.

This study was conducted as part of the broader Nomura study (Kawamoto, Tabara, Kohara, Miki, Kusunoki, et al., 2010). Participants were enrolled during their annual health checkups in 2002 in a rural area of Ehime Prefecture, Japan. The recruitment of participants was conducted systematically, as depicted in Figure 1. A total of 3164 residents were initially included in the sample. Plasma samples were collected after overnight fasting for all participants to measure HMW adiponectin levels. Due to limited plasma availability from certain participants, the sample size was reduced to 2056 individuals. Participants with missing values (38 men and 98 women) were excluded. Data on medical history, current health status, and medication use were gathered through interviews. This study analyzed data from the 2023 assessment cycle, which included a total of 1910 participants (845 men [61 ± 13; range, 20–89 years] and 1065 women [63 ± 11; range, 22–88 years]). Their residency status was verified using Japan's Basic Resident Ledger database, which provides detailed records of Japanese citizens and was used to confirm their survival or mortality status (December 2023). The research protocol was reviewed and approved by the Institutional Review Board (IRB) of Ehime University Hospital (approval number: 1903018). Informed written consent was obtained from all participants.

### Evaluation of risk factors

The study involved measuring participants' weight and height. Body mass index (BMI) was determined by dividing the weight in kilograms by the square of the height in meters. Smoking habits were assessed using pack years, calculated by multiplying the number of years a person smoked by the average number of packs smoked daily. Participants were categorized into four smoking groups: nonsmokers, former smokers, light smokers (fewer than 20 pack‐years), and heavy smokers (more than 20 pack‐years). Systolic blood pressure and diastolic blood pressure were measured twice on the right upper arm while participants were seated after a minimum of 5 min of rest. A properly sized cuff and an automatic oscillometric blood pressure recorder (BP‐103i; Colin, Aichi, Japan) were used, and the average of both readings was recorded. Blood samples were also collected to assess various biochemical markers such as triglycerides (TG), serum uric acid (SUA), fasting plasma glucose (FPG), creatinine (Cr), high‐density lipoprotein cholesterol (HDL‐C), low‐density lipoprotein cholesterol (LDL‐C), and HMW adiponectin. The estimated glomerular filtration rate (eGFR) was calculated using the Chronic Kidney Disease Epidemiology Collaboration (CKD‐EPI) equation, adjusted with coefficients specific to the Japanese population. For males with a serum Cr level of 0.9 mg/dL or lower, the formula was 141 × (Cr/0.9)^0^·^411^ × 0.993^age^ × 0.813. If Cr levels exceeded 0.9 mg/dL, the equation became 141 × (Cr/0.9)^1^·^209^ × 0.993^age^ × 0.813. Similarly, for females with Cr levels of 0.7 mg/dL or lower, the formula used was 144 × (Cr/0.7)^0^·^329^ × 0.993^age^ × 0.813, while for Cr levels above 0.7 mg/dL, the equation changed to 144 × (Cr/0.7)^1^·^209^ × 0.993^age^ × 0.813 (Horio et al., 2010).

The participants were grouped according to established criteria. Participants were considered hypertensive if their systolic blood pressure was at least 140 mmHg, their diastolic blood pressure was at least 90 mmHg, or if they were receiving antihypertensive therapy (Chobanian et al., 2003). Hypertriglyceridemia was defined as having TG levels of 150 mg/dL or higher. Individuals with HDL‐C levels of 40 mg/dL or lower were classified as having hypo‐HDL cholesterolemia. Participants were identified as having hyperlipidemia if their LDL‐C levels were 140 mg/dL or higher or if they were undergoing treatment with antidyslipidemic drugs (Teramoto et al., 2007). Those exhibiting FPG levels of 126 mg/dL or above and utilizing antidiabetic medication were designated as diabetic (Association AD, 2019). Hyperuricemia was defined by SUA levels of 7.0 mg/dL or higher, including those taking SUA‐lowering medications (Hisatome et al., 2021). Lastly, an eGFR below 60 mL/min/1.73 m^2^ was used as a chronic kidney disease (CKD) criterion (Chen et al., 2019). Participants were diagnosed with CVD if they had a history of conditions such as ischemic heart disease, peripheral vascular disease, or ischemic stroke at the start of the follow‐up period.

### Measurement of alcohol consumption and HMW adiponectin levels

Alcohol consumption was measured in sake units, where one unit corresponds to 22.9 g of ethanol. Participants were classified based on their drinking habits into nondrinkers, occasional drinkers (less than one unit per day), light daily drinkers (one to two units per day), and heavy daily drinkers (two to three units per day). No participant consumed more than three units daily. Plasma levels of HMW adiponectin were measured using a recently developed enzyme‐linked immunosorbent assay (ELISA) system (FUJIREBIO, Tokyo, Japan). This system utilizes a monoclonal antibody specific to HMW adiponectin, the details of which have been described previously. The intra‐ and interassay coefficients of variation for this adiponectin assay ranged from 2.1% to 7.3% and 6.3% to 7.2%, respectively, depending on the concentration of the substance being measured.

### Statistical analysis

All statistical analyses were conducted separately by gender using IBM SPSS Statistics Version 20 (Statistical Package for Social Science Japan, Inc., Tokyo, Japan). Unless otherwise specified, data are presented as the mean ± standard deviation (SD). For parameters with nonnormal distributions (TG, FPG, and HMW adiponectin), data are shown as the median (interquartile range). Nonnormally distributed variables were log‐transformed prior to analysis. Participants were divided into three groups within each gender, based on tertiles of HMW adiponectin. Differences between the groups were assessed using the Student's *t*‐test for continuous variables and the *χ* ^2^ test for categorical variables. A univariable analysis using the Cox proportional hazards model was conducted for each baseline characteristic, and all confounding factors were included as covariates. Additionally, a multivariable analysis was performed using the forced entry method, also based on the Cox proportional hazards model, with age as the primary time scale, with entry time defined as the subject's age at recruitment and exit time defined as the age at death or censoring (end of the follow‐up period). Hazard ratios (HRs) and 95% confidence intervals (CIs) were estimated through Cox proportional hazards regression. Subsequently, a likelihood ratio test was conducted to evaluate the interactions between alcohol consumption groups and HMW adiponectin variables. The interaction effect was analyzed while adjusting for all confounding factors. A *p*‐value of <0.05 was considered statistically significant.

## RESULTS

### Baseline characteristics by alcohol consumption

Table 1 shows the baseline characteristics of subjects according to alcohol consumption, which is known to influence HMW adiponectin levels [16–19]. Of the participants, 809 (42.4%) were defined as nondrinkers, 561 (29.4%) occasional drinkers, 346 (18.1%) daily light drinkers, and 194 (10.2%) daily heavy drinkers. The prevalence of heavy smoking, hypertriglyceridemia, and hyperuricemia was higher, but the prevalence of dyslipidemia and HMW adiponectin was lower in daily heavy drinkers than in non‐ and occasional drinkers. There were no intergroup differences regarding BMI, the prevalence of low‐HDL cholesterolemia, and the prevalence of diabetes. The higher the amount of alcohol consumed, the greater the proportion of men who drank heavily, and the same trend was observed among smokers. The history of CVD and the prevalence of hypertension were lower in occasional drinkers.

**TABLE 1:** Baseline characteristics of individuals according to alcohol consumption.

Characteristics	Alcohol consumption				*p* for trend *
	Non	Occasional	Daily light	Daily heavy	
*N* = 1910	*N* = 809	*N* = 561	*N* = 346	*N* = 194	
Gender (male), *n* (%)	117 (14.5)	243 (43.3)	296 (85.5)	189 (97.4)	**<0.001**
Age (years)	66 ± 10	59 ± 13	61 ± 13	59 ± 12	**<0.001**
Body mass index (kg/m^2^)	23.4 ± 3.2	23.4 ± 3.2	23.4 ± 3.0	23.9 ± 3.3	0.305
Smoking habits					
Non‐smoker, *n* (%)	730 (90.2)	424 (75.6)	178 (51.4)	69 (30.4)	**<0.001**
Past smoker, *n* (%)	52 (6.4)	94 (16.8)	120 (34.7)	80 (41.2)	
Light smoker, *n* (%)	13 (1.6)	17 (3.0)	25 (7.2)	21 (10.8)	
Heavy smoker, *n* (%)	14 (1.7)	26 (4.6)	23 (6.6)	34 (17.5)	
History of cardiovascular disease, *n* (%)	85 (10.5)	29 (5.2)	31 (9.0)	14 (7.2)	**0.005**
Hypertension, *n* (%)	493 (60.9)	246 (43.9)	187 (54.0)	127 (65.6)	**<0.001**
Systolic blood pressure (mmHg)	141 ± 23	134 ± 21	139 ± 21	144 ± 20	**<0.001**
Diastolic blood pressure (mmHg)	81 ± 12	80 ± 12	83 ± 11	87 ± 11	**<0.001**
Antihypertensive medication, *n* (%)	264 (32.6)	107 (19.1)	89 (25.7)	46 (23.7)	**<0.001**
Dyslipidemia, *n* (%)	294 (36.3)	157 (28.0)	72 (20.8)	24 (12.4)	**<0.001**
LDL‐cholesterol (mg/dL)	128 ± 29	118 ± 31	109 ± 30	100 ± 32	**<0.001**
Antilipidemic medication, *n* (%)	55 (6.8)	31 (5.5)	14 (4.0)	4 (2.1)	**0.037**
Low‐HDL cholesterolemia, *n* (%)	40 (4.9)	36 (6.4)	15 (4.3)	9 (4.5)	0.490
HDL cholesterol (mg/dL)	62 ± 15	62 ± 16	62 ± 15	65 ± 17	0.097
Hypertriglyceridemia, *n* (%)	147 (18.2)	89 (15.9)	62 (17.9)	49 (25.3)	**0.035**
Triglycerides (mg/dL)	94 (72–133)	66 (89–122)	89 (68–126)	107 (77–151)	**<0.001**
Diabetes, *n* (%)	62 (7.7)	41 (7.3)	23 (6.6)	14 (7.2)	0.946
Fasting plasma glucose (mg/dL)	93 (88–101)	92 (87–100)	95 (89–102)	98 (90–108)	**<0.001**
Antidiabetic medication, *n* (%)	36 (4.4)	21 (3.7)	7 (2.0)	4 (2.1)	0.135
Chronic kidney disease, *n* (%)	90 (11.1)	40 (7.1)	23 (6.6)	18 (9.3)	**0.026**
eGFR (mL/min/1.73 m^2^)	78.1 ± 18.0	81.8 ± 17.1	80.9 ± 15.4	83.8 ± 16.8	**<0.001**
Hyperuricemia, *n* (%)	30 (3.7)	73 (13.0)	86 (24.9)	72 (37.1)	**<0.001**
Serum uric acid (mg/dL)	4.6 ± 1.1	5.1 ± 1.4	5.8 ± 1.4	6.3 ± 1.4	**<0.001**
HMW adiponectin (μg/mL)	6.44 (4.13–9.72)	5.03 (2.94–7.58)	3.77 (2.33–6.47)	2.90 (1.81–4.34)	**<0.001**
1st SD, *n* (%)	65 (8.0)	98 (17.5)	87 (25.1)	78 (40.2)	**<0.001**
2nd SD, *n* (%)	202 (25.0)	165 (29.4)	133 (38.4)	76 (39.2)	
3rd SD, *n* (%)	342 (42.3)	229 (40.8)	87 (25.1)	37 (19.1)	
4th SD, *n* (%)	200 (24.7)	69 (12.3)	39 (11.3)	3 (1.5)	

*Note*: Data presented as mean ± standard deviation. Data for triglycerides, fasting plasma glucose, and HMW adiponectin were skewed, presented as median (interquartile range) values, and log‐transformed for analysis. Significant values (*p* < 0.05) are presented in bold

Abbreviations: eGFR, estimated glomerular filtration ratio; HDL, high‐density lipoprotein; HMW, high molecular weight; LDL, low‐density lipoprotein.

*p‐value from Student's *t*‐test for the continuous variables or from *χ* ^2^‐test for the categorical variables.

### Kaplan–Meier survival curves of HMW adiponectin categories for all‐cause mortality by alcohol consumption

**FIGURE 2:**
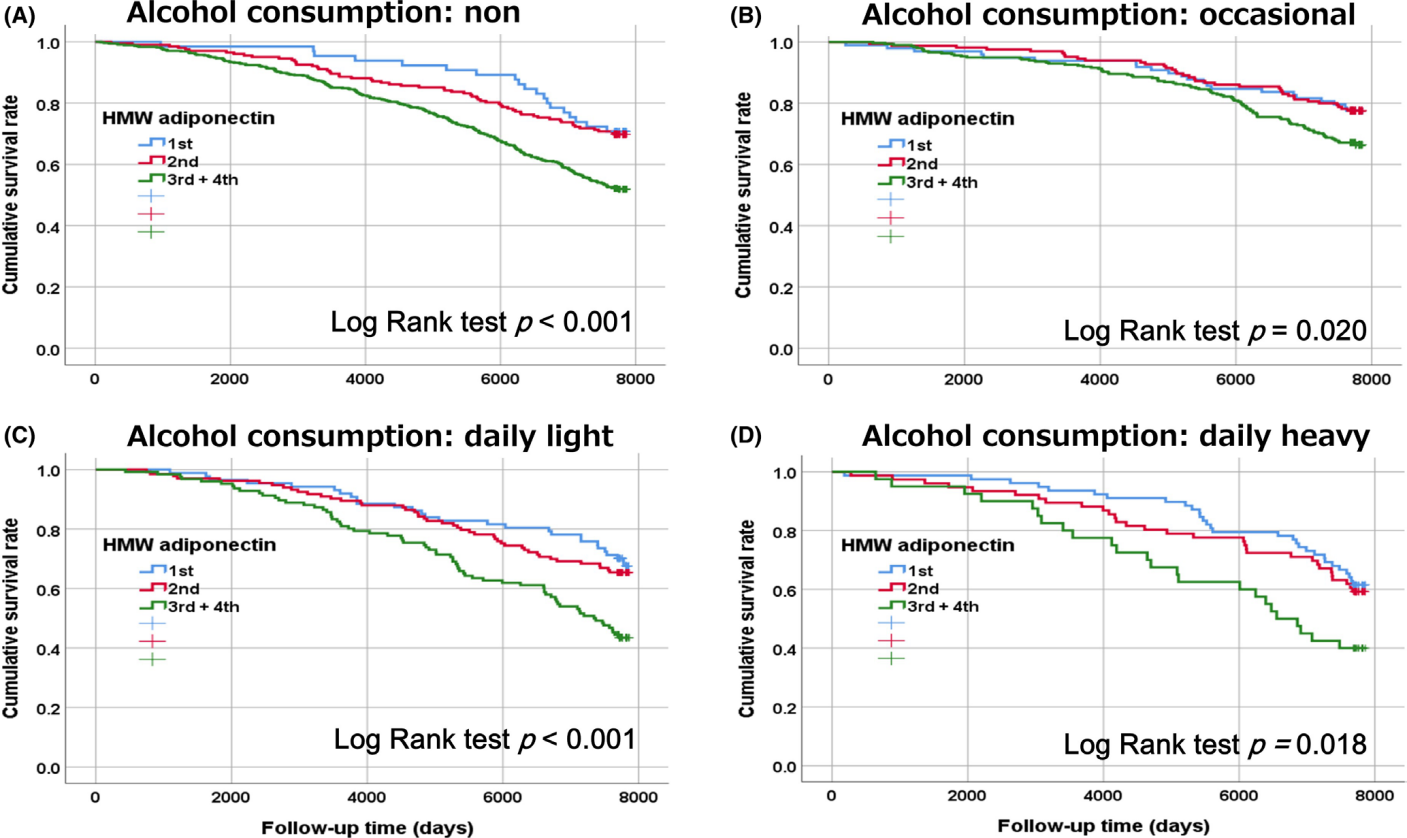
Kaplan–Meier survival curves for overall survival of subjects categorized by BMI and HOMA‐IR. Participants were divided into two groups based on a BMI of either above or below 22.0 kg/m^2^ and into four groups by one standard deviation of HOMA‐IR. (A), nondrinker; (B), occational drinker; (C), daily light drinker; (D) daily heavy drinker.

The median duration of follow‐up was 7706 days, with an interquartile range of 6080 to 7762 days. Of all participants, 727 individuals (38.1%) were confirmed to have died. This included 378 men, representing 44.7% of the male cohort, and 349 women, accounting for 32.8% of the female cohort. Over the follow‐up period, 340 deaths (42.0%) occurred among nondrinkers, 158 (28.2%) among occasional drinkers, 144 (41.6%) among daily light drinkers, and 85 (43.8%) among daily heavy drinkers, regardless of cause. Kaplan–Meier survival estimates were plotted to compare survival rates across different HMW adiponectin categories, stratified by drinking habits: nondrinkers, occasional drinkers, daily light drinkers, and daily heavy drinkers (Figure 2). The results indicated that survival rates were significantly lower among individuals in the highest HMW adiponectin category, irrespective of alcohol consumption. However, within the highest HMW adiponectin category, occasional drinkers exhibited lower survival rates than other groups.

### HRs and 95% CIs of baseline characteristics for all‐cause mortality

Table 2 presents the HRs for mortality associated with various confounders, including HMW adiponectin. In addition to sex, age, smoking habits, history of CVD, diabetes, and hyperuricemia, individuals who either abstained from alcohol (HR, 1.23; 95% CI, 1.00–1.52) or engaged in daily heavy drinking (HR, 1.39; 95% CI, 1.04–1.86) exhibited significantly higher overall mortality compared with occasional drinkers (reference) who exhibited the most significant difference between the two categories in the univariable analysis. Furthermore, individuals with the 3rd SD level (HR, 1.39; 95% CI, 1.07–1.80) and 4th SD level of HMW adiponectin (HR, 1.65; 95% CI, 1.23–2.23) had a significantly increased risk of all‐cause mortality compared to those with the lowest levels. In this study, the 3rd and 4th SDs demonstrated similarly significant hazard ratios compared to the 1st SD. Therefore, the 3rd and 4th SDs were combined for analysis.

**TABLE 2:** Hazard ratios (HR) and 95% confidence intervals (CI) of baseline characteristics for all‐cause mortality.

Baseline characteristics *N* = 1910	Non‐adjusted HR (95% CI)	Adjusted HR (95% CI)
Gender (male vs. female = 1)	**0.67 (0.58–0.77)**	**0.63 (0.50–0.80)**
Age (per 1 year)	**1.13 (1.12–1.14)**	**1.12 (1.11–1.13)**
Body mass index (per 1 kg/m^2^)	**0.96 (0.94–0.98)**	0.98 (0.95–1.00)
Smoking habits (non = 0/past = 1/light = 2/heavy = 3) (per 1)	**1.26 (1.16–1.37)**	**1.14 (1.03–1.25)**
Drinking habits (occasional = 0 vs. non = 1)	**1.67 (1.38–2.01)**	**1.23 (1.00–1.52)**
Drinking habits (occasional = 0 vs. daily light = 1)	**1.63 (1.30–2.05)**	1.15 (0.91**–**1.47)
Drinking habits (occasional = 0 vs. daily heavy = 1)	**1.71 (1.31–2.22)**	**1.39 (1.04–1.86)**
History of cardiovascular disease	**3.00 (2.45–3.67)**	**1.39 (1.13–1.72)**
Hypertension	**2.44 (2.08–2.87)**	1.08 (0.91–1.29)
Hypertriglyceridemia	0.88 (0.72–1.07)	0.96 (0.78–1.19)
Low‐HDL cholesterolemia	1.05 (0.76–1.43)	0.86 (0.61–1.22)
High‐LDL cholesterolemia	1.00 (0.85–1.18)	0.96 (0.81–1.14)
Diabetes	**1.70 (1.34–2.16)**	**1.71 (1.34–2.19)**
Chronic kidney disease	**2.65 (2.18–3.23)**	1.16 (0.94–1.44)
Hyperuricemia	**1.50 (1.24–1.81)**	**1.42 (1.14–1.76)**
HMW adiponectin (1st SD = 0 vs. 2nd SD = 1)	1.05 (0.82–1.34)	1.08 (0.84–1.39)
HMW adiponectin (1st SD = 0 vs. 3rd SD = 1)	**1.52 (1.21–1.91)**	**1.39 (1.07–1.80)**
HMW adiponectin (1st SD = 0 vs. 4th SD = 1)	**2.27 (1.77–2.92)**	**1.65 (1.23–2.23)**

*Note*: The model was adjusted for gender, age, body mass index, smoking habits, drinking habits, history of cardiovascular disease, hypertension, hypertriglyceridemia, low‐HDL cholesterolemia, high‐LDL cholesterolemia, diabetes, chronic kidney disease, and hyperuricemia. Significant values (*p* < 0.05) are presented in bold.

Abbreviations: CI, confidence interval; HR, hazard ratio; SD, standard deviation.

### HRs and 95% CIs of HMW adiponectin categories for all‐cause mortality by alcohol consumption

Table 3 presents the HRs for HMW adiponectin levels, as both categorical and continuous variables, in relation to all‐cause mortality across groups stratified by alcohol consumption. After adjusting for all confounding factors, the HR for individuals with the highest HMW adiponectin levels was significantly elevated in the nondrinkers (HR, 1.89; 95% CI, 1.09–3.29), occasional drinkers (HR, 1.84; 95% CI, 1.05–3.21), and daily heavy drinkers (HR, 1.90; 95% CI, 1.05–3.44), but not in the group that consumed alcohol lightly daily. Additionally, a 1‐natural‐log‐unit increase in HMW adiponectin was associated with a significantly higher HR for all‐cause mortality in nondrinkers (HR, 2.33; 95% CI, 1.48–3.67) and occasional drinkers (HR, 2.56; 95% CI, 1.29–5.07), but not in daily light or heavy drinkers. The interaction between drinking habits and HMW adiponectin levels was significantly associated with all‐cause mortality.

**TABLE 3:** Hazard ratios (HR) and 95% confidence intervals (CI) of HMW adiponectin categories for all‐cause mortality according to alcohol consumption.

Baseline characteristics	Adjusted HR (95% CI)					*p* for interaction
	Total	Non	Occasional	Daily light	Daily heavy	
	*N* = 1910	*N* = 809	*N* = 561	*N* = 346	*N* = 194	
Death, *n* (%)	727 (38.1)	340 (42.0)	158 (28.2%)	144 (41.6)	85 (43.8)	
HWM adiponectin						
1st SD	Reference	Reference	Reference	Reference	Reference	
2nd SD	1.07 (0.83–1.38)	1.55 (0.86–2.76)	1.11 (0.63–1.93)	1.11 (0.68–1.82)	0.77 (0.45–1.31)	
3rd +4th SD	**1.42 (1.10–1.83)**	**1.89 (1.09–3.29)**	**1.84 (1.05–3.21)**	1.18 (0.71–1.97)	**1.90 (1.05–3.44)**	
*p* for trend	**0.003**	**0.049**	**0.023**	0.806	**0.012**	**0.027**
Continuous (per 1)	**1.91 (1.42–2.57)**	**2.33 (1.48–3.67)**	**2.56 (1.29–5.07)**	1.18 (0.61–2.27)	2.46 (0.96–6.30)	
*p*‐value	**<0.001**	**<0.001**	**0.007**	0.619	0.061	**0.005**

*Note*: HMW adiponectin values were skewed and log‐transformed for analysis. The model was adjusted for age and gender, drinking habits (total group), body mass index, smoking status, history of cardiovascular disease, hypertension, hypertriglyceridemia, low‐HDL cholesterolemia, high‐LDL cholesterolemia, diabetes, chronic kidney disease, and hyperuricemia. Significant values (*p* < 0.05) are presented in bold.

## DISCUSSION

A major finding of the present study was that alcohol consumption and increased HMW adiponectin were interactively and prospectively associated with all‐cause mortality among community‐dwelling persons. Participants who either abstained from alcohol (HR, 1.23; 95% CI, 1.00–1.52) or engaged in daily heavy drinking (HR, 1.39; 95% CI, 1.04–1.86) exhibited significantly higher overall mortality than occasional drinkers. Additionally, all‐cause mortality was significantly higher in individuals with elevated levels of HMW adiponectin than those with lower levels; however, no such relationship was observed in daily light drinkers. To our knowledge, this is the first study to indicate interactive associations of alcohol consumption and HMW adiponectin with all‐cause mortality in about 2000 Japanese community‐dwelling persons.

The association between high‐HMW adiponectin and mortality remains somewhat inconsistent. Although HMW adiponectin appears to exert protective effects against the initiation and progression of atherosclerotic lesions (Kobashi et al., 2005), population‐based studies investigating its role in CVD have produced mixed findings. Some studies indicate elevated HMW adiponectin levels are associated with a lower risk of CHD (Horáková et al., 2015; Pischon et al., 2011; Wang et al., 2013). In contrast, others showed U‐shaped relationships with CVD (Kizer et al., 2013) or no relationship with the risk for incident CHD (Sattar et al., 2008). Adiponectin—a protein known for its insulin‐sensitizing, antiinflammatory, antiatherogenic, and cardioprotective factors in cellular and animal models (Ebrahimi‐Mamaeghani et al., 2015)—is also associated with an elevated risk of CVD‐related and all‐cause mortality; this phenomenon is known as the adiponectin paradox and represents a case of “reverse epidemiology” (Beatty et al., 2012).

Two principal hypotheses have been proposed to explain this phenomenon. The first suggests that adiponectin acts as a biomarker for CVD severity, with increased adiponectin secretion in advanced CHD as an adaptive, protective response. This compensatory mechanism aims to counter inflammation, atherosclerosis, and hypoxia‐reoxygenation injuries while promoting angiogenesis in ischemic tissues (Hascoet et al., 2013). Supporting this hypothesis, Beatty et al. (2012) observed that the link between elevated adiponectin levels and mortality lost significance after adjusting for CHD severity. However, our findings did not support this hypothesis, as elevated HMW adiponectin levels were associated with mortality even after adjusting for confounding factors and history of CVD. The second hypothesis, supported by animal models, introduces the concept of adiponectin resistance—studies in humans, such as those by Van Berendoncks et al. (2010), have shown that despite increased adiponectin expression in skeletal muscle, heart failure patients exhibit down‐regulated adiponectin receptors, suggesting functional adiponectin resistance (Engin, 2024). Such resistance, potentially affecting vascular and cardiac tissues, may explain the association between elevated adiponectin levels and poor outcomes.

The interaction between adiponectin and alcohol consumption on mortality may be influenced by adiponectin's metabolic and cardiovascular protective effects, as well as by the metabolic changes and cardiovascular risks associated with alcohol intake. Moderate alcohol consumption has been shown to reduce cardiovascular risk, while excessive alcohol intake can promote inflammation, impair liver function, elevate blood pressure, and thereby increase cardiovascular and overall mortality risk (Piano, 2017). Alcohol consumption has also been suggested to affect adiponectin levels; for instance, moderate‐to‐heavy alcohol intake may decrease adiponectin levels (Kawamoto, Tabara, Kohara, Miki, Ohtsuka, et al., 2010) or increase adiponectin levels (Imhof et al., 2009). Conversely, excessive alcohol consumption may impact adiponectin receptors, diminishing the effectiveness of adiponectin and potentially inducing “adiponectin resistance,” which may adversely affect cardiovascular risk and mortality.

Our study possesses several strengths, including a long‐term follow‐up period and adjustments for potential confounders. However, certain limitations should be acknowledged. First, the cohort study design does not fully establish causality between baseline characteristics and all‐cause mortality. Second, we adopted a cohort design, evaluating baseline characteristics such as alcohol consumption and HMW adiponectin levels at the initial visit. However, it is crucial to acknowledge that alcohol consumption, HMW adiponectin levels, and certain covariates may vary over time, potentially shifting during a prolonged follow‐up period. As a result, the study's findings could be more likely to be underestimated than overestimated due to nondifferential misclassification bias. Third, we could not completely account for the influence of underlying conditions or medications for hypertension, dyslipidemia, diabetes, and hyperuricemia, among others, which may limit the generalizability of our findings due to specific demographic and referral characteristics. Fourth, our study utilized Japan's Basic Resident Register to track all deaths, regardless of cause. However, this method may have excluded individuals who emigrated during the survey period. Employing all‐cause mortality as the outcome is regarded as a suitable method for comprehensively assessing the overall influence of health risk factors using reliable data. Lastly, the relatively small number of participants and mortality events may reduce the strength of the observed association between HMW adiponectin levels and all‐cause mortality.

## CONCLUSION

Alcohol consumption was associated with all‐cause mortality in a U‐shaped manner, whereas adiponectin levels were associated with mortality in a dose‐dependent fashion. Additionally, an interaction between alcohol consumption and adiponectin levels was observed, independent of traditional cardiovascular risk factors. Prospective population‐based studies are required to better understand the mechanisms underlying this association in healthy, community‐dwelling individuals. Such studies will help determine whether interventions, including effective lifestyle modifications that influence HMW adiponectin levels in adults, could reduce mortality risk.

## AUTHOR CONTRIBUTIONS

RK and AK contributed to the study design, performed the statistical analysis, and drafted the manuscript. RK, AK, MA, and DN were responsible for the acquisition and interpretation of data. RK also contributed to the conceptualization and design of the statistical analysis. All authors have read and approved the final manuscript.

## CONFLICT OF INTEREST STATEMENT

The authors declare no competing interests.

## Data Availability

The data supporting the findings of this study were obtained from the Ethics Committee of Ehime University Hospital. Due to licensing restrictions, the data are not publicly available. However, the data can be made available from the corresponding author upon reasonable request and with permission from the Ethics Committee of Ehime University Hospital.
